# Folding paper models of biostructures for outreach and education

**DOI:** 10.1016/j.patter.2024.100931

**Published:** 2024-02-09

**Authors:** David S. Goodsell, Shuchismita Dutta, Brian P. Hudson, Maria Voigt, Stephen K. Burley, Christine Zardecki

**Affiliations:** 1 Research Collaboratory for Structural Bioinformatics Protein Data Bank, Institute for Quantitative Biomedicine, Rutgers, The State University of New Jersey, Piscataway, NJ 08854, USA; 2 Rutgers Cancer Institute of New Jersey, New Brunswick, NJ 08901, USA; 3 The Scripps Research Institute, La Jolla, CA 92037, USA; 4 Research Collaboratory for Structural Biology Protein Data Bank, San Diego Supercomputer Center, University of California San Diego, La Jolla, CA 92093, USA; 5 Department of Chemistry and Chemical Biology, Rutgers, The State University of New Jersey, Piscataway, NJ 08854, USA

## Abstract

Molecular origami offers an offline way to explore the 3D structures of biology. Visit PDB101.rcsb.org to download free paper models of DNA, green fluorescent protein, viruses, and more.

## Main text

Structural studies provide unique windows into the biological world, revealing at the atomic level the molecular mechanisms that orchestrate the processes of life. Biomolecular structure can be daunting, however, with its incredible complexity. The Research Collaboratory for Structural Bioinformatics Protein Data Bank (RCSB PDB, RCSB.org)^1^^,^^2^ is always looking for ways to make biomolecules familiar and comprehensible to experts and non-experts alike. At PDB-101 (PDB101.RCSB.org), RCSB PDB’s training and outreach portal, we offer many modalities for learning, including monthly feature articles, animations, posters, and lesson plans.^3^ As part of this effort, we strive to develop compelling hands-on activities in classrooms and outreach events that will promote an understanding of biological macromolecules in 3D. To reach a wide audience, these activities need to be easily accessible and at near-zero cost. We have found that printable paper models or “molecular origami” fit these parameters.

**Figure 1:**
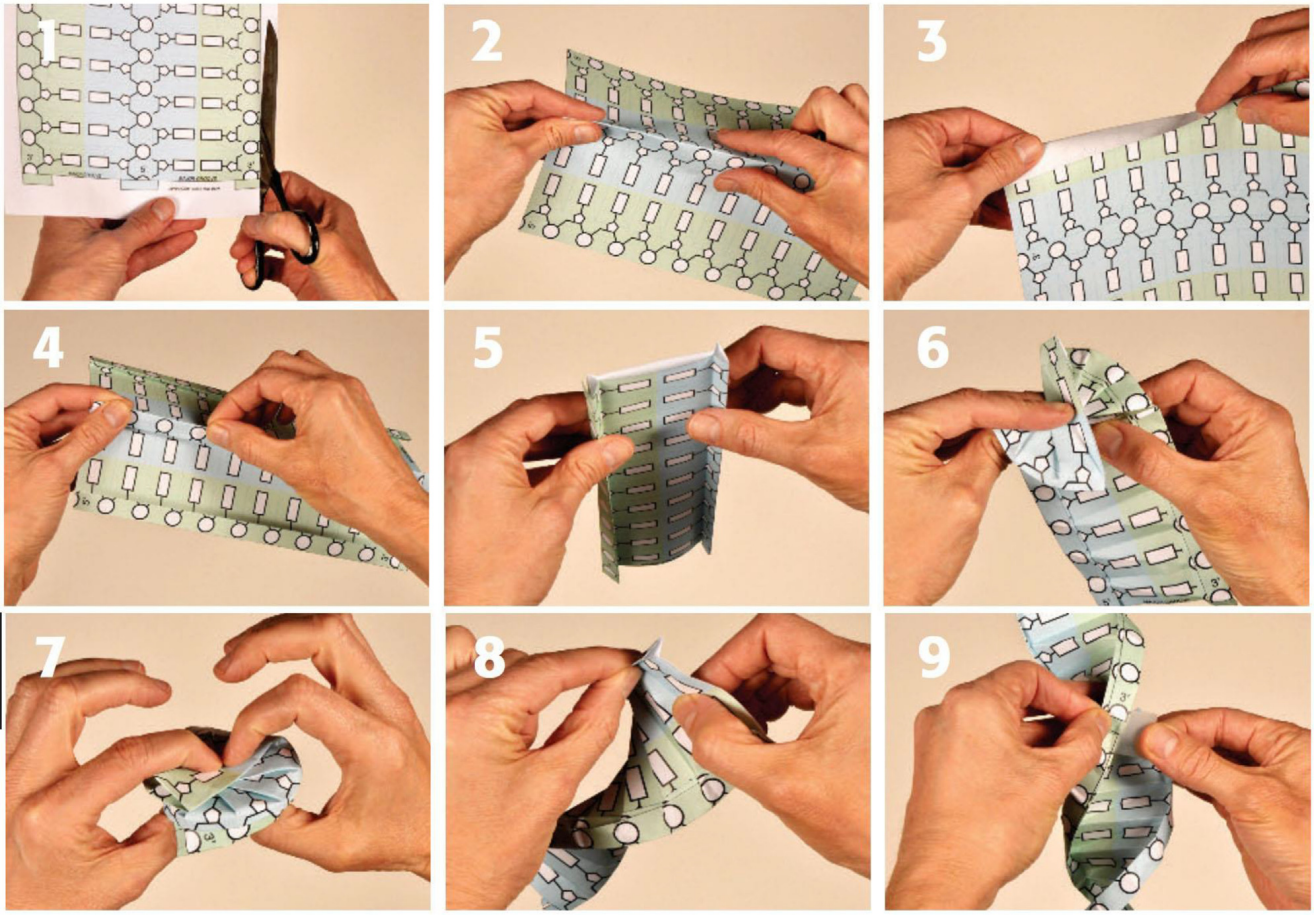
Step-by-step pictorial instructions for folding a paper model of DNA Students can color in the bases (small rectangular boxes between the backbones) or add nucleotide names to customize their model.

Our collection started with a model of the most celebrated biomolecule: double-stranded B-form DNA.^4^ In our discussions, educators almost invariably ask for engaging materials about genetic mechanisms, so focusing on DNA was an ideal place to start. The model uses a traditional origami approach where a single sheet of paper is folded into the shape of the iconic right-handed double helix. Step-by-step folding instructions (Figure 1) and tutorial videos are available in English and Spanish. Other DNA paper models exist online at other resources; the PDB-101 version offers supporting materials and links to related content. The sheet is printed with a schematic representation of the three parts of each nucleotide: sugar, phosphate, and base. Each part is left intentionally blank to provide an opportunity to color and design different base sequences. To deepen discussions with students during a folding activity, the PDF also includes a second foldable template (not shown) that includes detailed depictions of atomic structures for each nucleotide. The DNA model is our most popular molecular origami offering (downloaded ∼3,000 times annually).

**Figure 2:**
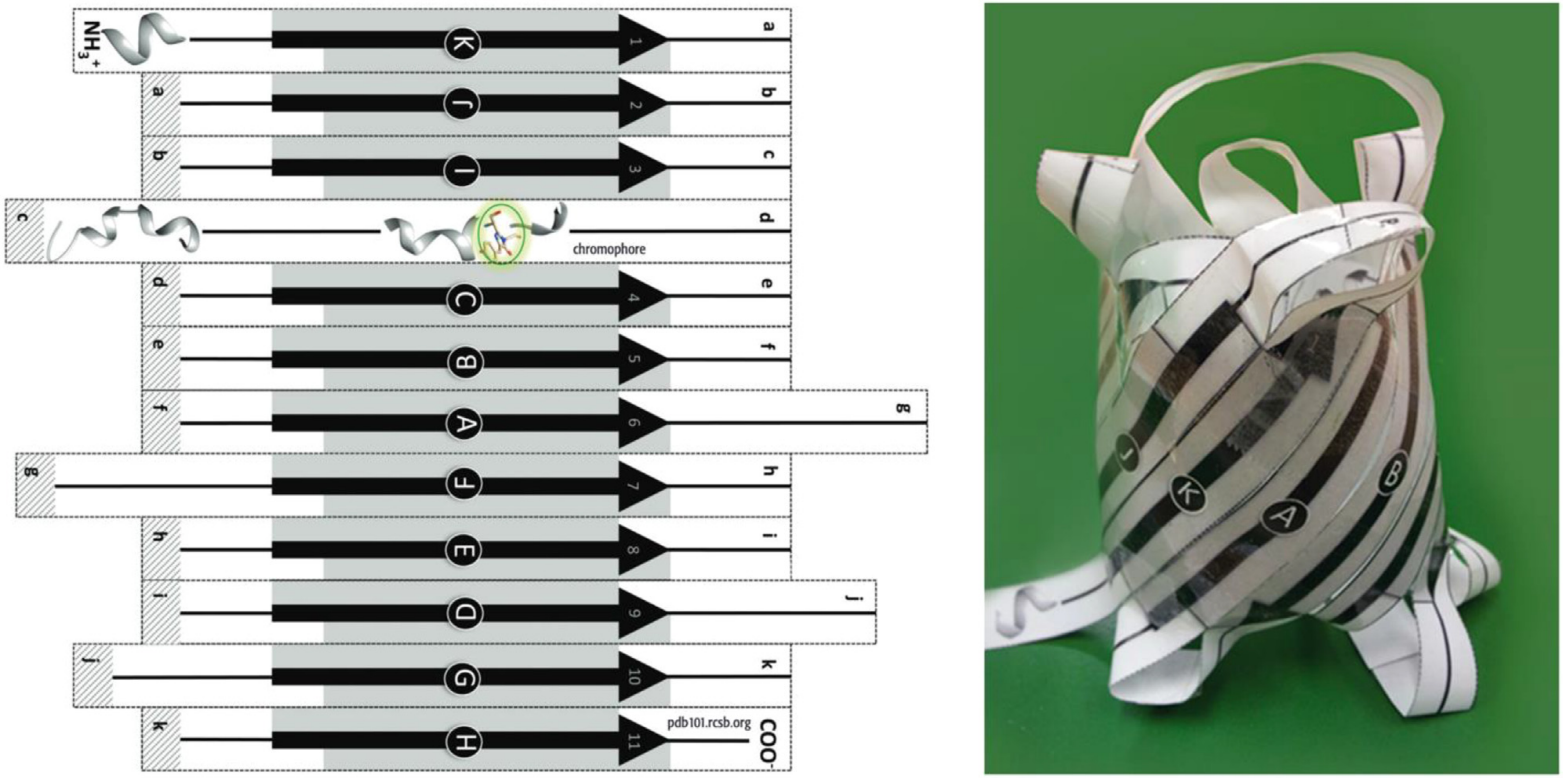
Paper model of green fluorescent protein Users print the template (left), cut along dotted lines, tape pieces into one long strand, and match up beta strands (black arrows) in alphabetical order to create the beta barrel structure (right).

Protein folding is another key foundational concept for 3D understanding of biological processes. We have created several models that provide a tangible experience in understanding the hierarchical process of folding a protein chain first into local secondary structures and then into functional tertiary structures. Figure 2 shows a paper model of green fluorescent protein (GFP), a protein often used in classroom settings. In the activity, students first cut out the strands and tape them into one long strip representing the unfolded primary structure of the polypeptide chain. Regions of secondary structure, in this case beta strands, are denoted schematically on this strip. The students then form loops between the beta strand regions and tape neighboring strands together to form the folded beta barrel of GFP. An online activity augments the physical model, providing an interactive computer-graphics view of the atomic structure for comparison with the model. Additional paper models available at PDB-101 allow students to explore other folding patterns, including a G-protein-coupled receptor, an immunoglobulin G antibody, insulin, and a model that explores the trefoil fold of transfer RNA.

**Figure 3:**
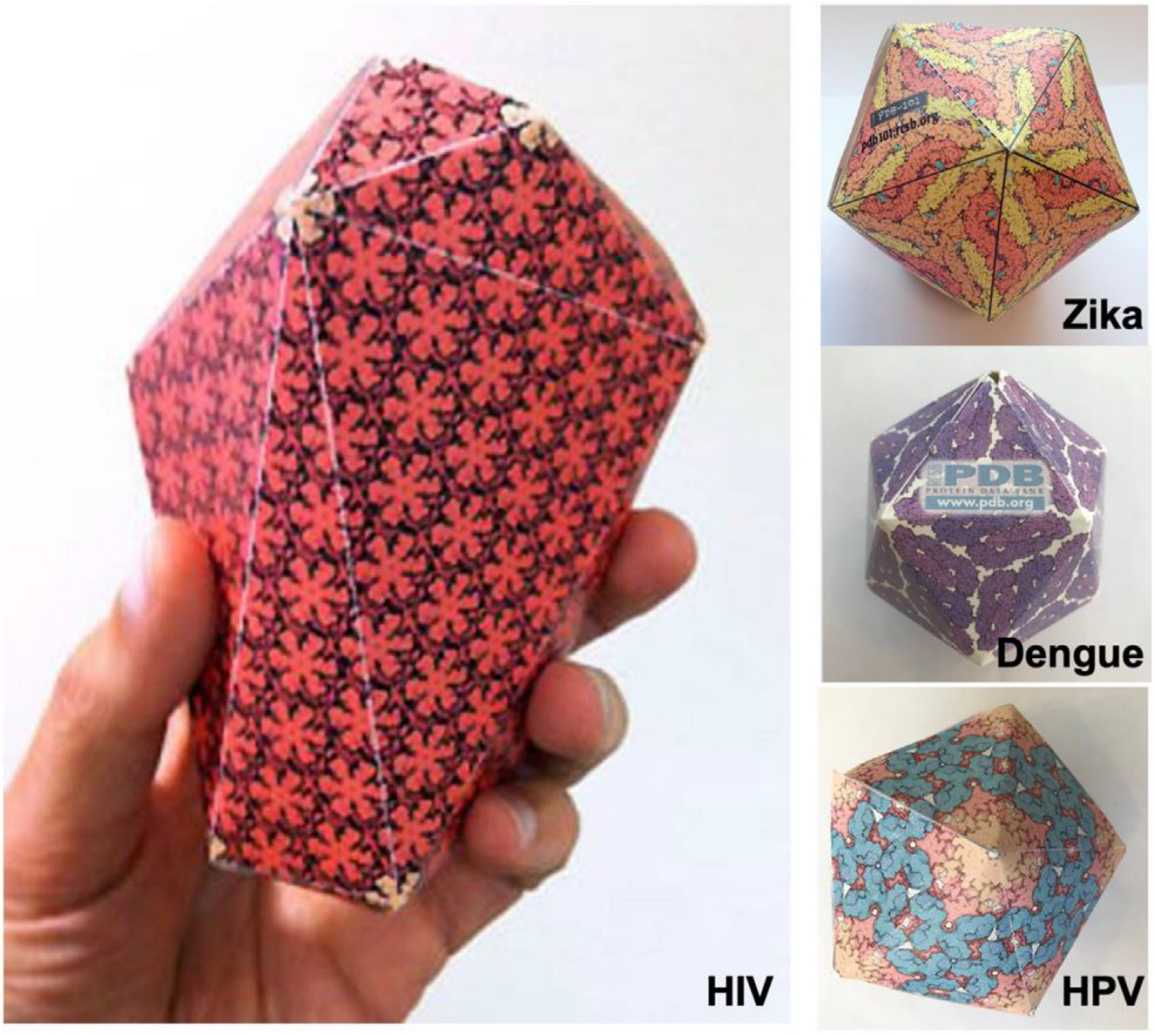
Paper models of virus capsids created in response to public health threats

We have also created paper models highlighting protein structures related to public health challenges, including HIV, Dengue virus, and its close relative Zika virus (Figure 3). A paper model of human papilloma virus (HPV), a common cause of genital and oral cancers, is also available (Figure 3). These models provide teachers with a user-friendly activity for furthering understanding of viruses in 3D and discussing health threats. These models have been expanded into an advanced collection highlighting viral quasisymmetry, a difficult topic often only presented at the graduate level. Molecular origami models allow students to explore how, through small deformations, a single type of viral protein can be used to create much larger icosahedral capsids in different viruses.

In keeping with RCSB PDB’s long-standing commitment to FAIR (findable, accessible, interoperable, and reusable) principles, all models are freely available at PDB-101 along with supplemental materials and links to corresponding experimental data. Paper models are provided as printable PDF files for easy use with minimal technological requirements. We have also printed customized punch-out templates for many of the virus models. Both have been invaluable at science festivals, professional scientific society meetings, and science education conferences.

To learn (and fold) more, please visit PDB-101 at https://pdb101.rcsb.org/learn/paper-models.

## About the authors

When not thinking about molecules in origami form, RCSB PDB members take different approaches in promoting structural views of biology. **David S. Goodsell** paints molecular landscapes in watercolor and other media. **Shuchismita Dutta** and **Stephen K. Burley** lead an undergraduate honors seminar course that explores the molecular anatomy of health and disease. **Brian P. Hudson** expertly curates and rigorously validates data in collaboration with PDB depositors. **Maria Voigt** creates detailed 3D molecular animations that demonstrate how PDB structures function in the cell. **Christine Zardecki** promotes all RCSB PDB activities on social media and beyond. Learn more at https://RCSB.org.
